# A proof of concept for a deep learning system that can aid embryologists in predicting blastocyst survival after thaw

**DOI:** 10.1038/s41598-022-25062-z

**Published:** 2022-12-07

**Authors:** P. Marsh, D. Radif, P. Rajpurkar, Z. Wang, E. Hariton, S. Ribeiro, R. Simbulan, A. Kaing, W. Lin, A. Rajah, F. Rabara, M. Lungren, U. Demirci, A. Ng, M. Rosen

**Affiliations:** 1 Center for Reproductive Health, Department of Medicine, University of California, San Francisco, USA; 2 Department of Computer Science, Stanford University, Stanford, USA; 3 Center for Artificial Intelligence in Medicine & Imaging, Stanford University, Stanford, USA; 4 Canary Center for Cancer Early Detection, Stanford University, Stanford, USA

**Keywords:** Medical research, Predictive markers

## Abstract

The ability to understand whether embryos survive the thaw process is crucial to transferring competent embryos that can lead to pregnancy. The objective of this study was to develop a proof of concept deep learning model capable of assisting embryologist assessment of survival of thawed blastocysts prior to embryo transfer. A deep learning model was developed using 652 labeled time-lapse videos of freeze–thaw blastocysts. The model was evaluated against and along embryologists on a test set of 99 freeze–thaw blastocysts, using images obtained at 0.5 h increments from 0 to 3 h post-thaw. The model achieved AUCs of 0.869 (95% CI 0.789, 0.934) and 0.807 (95% CI 0.717, 0.886) and the embryologists achieved average AUCs of 0.829 (95% CI 0.747, 0.896) and 0.850 (95% CI 0.773, 0.908) at 2 h and 3 h, respectively. Combining embryologist predictions with model predictions resulted in a significant increase in AUC of 0.051 (95% CI 0.021, 0.083) at 2 h, and an equivalent increase in AUC of 0.010 (95% CI −0.018, 0.037) at 3 h. This study suggests that a deep learning model can predict in vitro blastocyst survival after thaw in aneuploid embryos. After correlation with clinical outcomes of transferred embryos, this model may help embryologists ascertain which embryos may have failed to survive the thaw process and increase the likelihood of pregnancy by preventing the transfer of non-viable embryos.

## Introduction

Over the past decade there has been a progressive increase in the number of frozen embryo transfer (FET) cycles; currently accounting for more than 50% of embryo transfers in assisted reproduction centers^1^. This increase is likely multifactorial, including increased pre-implantation genetic testing for aneuploidy (PGT-A)^2^, the promotion of elective single embryo transfers to reduce risk of multiple gestation, and increasingly the return for utilization of embryos previously frozen for fertility preservation^3–5^. As these circumstances are unlikely to change in the near future**,** improved understanding of how to discern the reproductive viability of a freeze–thaw embryo will become of increasing importance.

There have been several techniques used to select the best frozen embryo for transfer, including morphologic grading, morphokinetics, blastocyst culture, and PGT-A^6^. More recently, artificial intelligence methods that analyzed images of fresh blastocysts have been shown to be able to generate consistent quality scoring^7^. These tools have all been tailored towards predicting birth outcomes based on fresh embryo development but fail to adequately account for the mediating factor of possible freeze–thaw damage. It is assumed that almost every thawed embryo (> 95%) survives at point of initial evaluation, however, the literature surrounding how to assess for survival of a freeze–thaw embryo is limited as we have not previously studied embryo survival, hence we are likely overestimating survival^8^. Prior studies suggesting resumption of mitosis or intact blastomeres as assessment criteria are somewhat outdated, as the studies were completed on day 3 embryos frozen by mostly slow freeze methods^6,9^ A more recent publication assessing birth outcomes based on post-thaw blastocyst characteristics found that proportion of intact cells after thaw was predictive of birth outcomes, but the authors admitted judgement of this proportion was subjective^10^. Ultimately, improved discernment of factors that define viability of thawed embryos may help to predict their implantation potential.

Evaluating the viability of an embryo post-thaw is limited by the time constraint of needing to transfer that embryo into a patient, as most labs thaw embryos the morning of the FET for transfer 2 to 3 h later. Therefore, the judgement regarding thawed embryo implantation potential and subsequent decision whether to thaw additional embryos for transfer must be made within that short time frame. Establishing a rigorous evaluation process for embryos during this critical period may help to clarify whether freeze–thaw damage is a potential explanation of unexpectedly poor outcomes from embryos deemed good quality based on assessments made prior to cryopreservation. Furthermore, reliably identifying embryos that did not survive the thaw prior to transfer, beyond our current time-limited visual evaluation, would allow clinics to thaw further embryo or cancel the transfer, depending on the clinical situation.

The objective of this proof-of-concept study was to investigate if a deep learning model could assess and improve on embryologists’ assessment of survival of freeze–thaw blastocysts on images captured up to 3 h post-thaw. Given the real-world time constraints of needing to transfer an embryo soon after thaw, our model was trained on clinically relevant time points between 0- and 3-h post-thaw and its performance was compared to embryologist predictions of blastocyst survival after 25 h in culture post-thaw.

## Results

Of the 652 embryos in the dataset, 100% survived at 0, 2, and 3 h. There were 461 (70.7%) embryos that survived thaw at 25 h, and 191 (29.3%) that did not. The mean (SD) age of patients at the time of embryo creation that survived was 35.0 (6.0) years, and of those that did not was 36.8 (4.71) years. This difference was statistically significant (p < 0.01) and its implications are presented in the discussion. The training set contained 463 embryos (from 84 patients; 366 survived, 97 not survived). The validation set contained 90 videos of embryos (from 17 patients; 45 survived, 45 not survived. The test set contained 99 videos of embryos (from 18 patients; 50 survived; 49 not survived).

### Model performance

The ensemble model (EmbryoNeXt) achieved the highest AUC of 0.869 (95% CI 0.789, 0.934) with predictions made at 2 h, outperforming all other models at all different time points. Of the individual models in the ensemble at 2 h, ResNet50 had the highest AUC of 0.831 (95% CI 0.742, 0.902) at 2 h, followed by ResNet34 with an AUC of 0.826 (95% CI 0.742, 0.897). ResNet18 had an AUC of 0.791 (95% CI 0.701, 0.872), and DenseNet121 had the lowest AUC of 0.738 (95% CI 0.636, 0.836).

**Figure 1:**
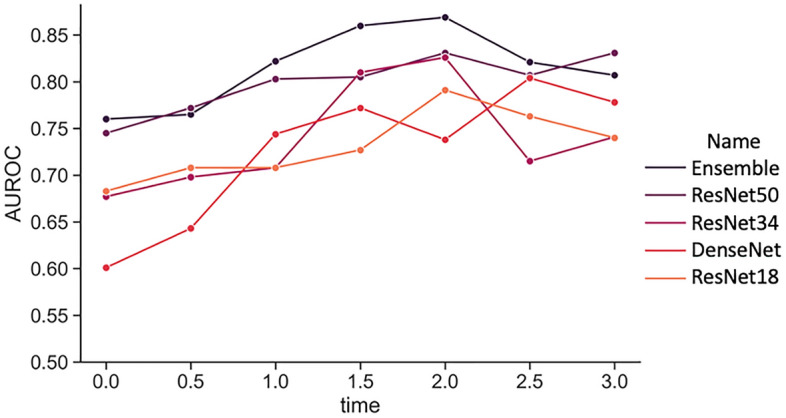
Model performances. Performance of different model architectures (AUROC) and the ensemble model (EmbryoNeXt) at different time points in the embryo thaw process (hrs).

The performance of the ensemble increased from an AUC of 0.76 (95% CI 0.664, 0.847) with predictions made at 0 h to 0.822 (95% CI 0.733, 0.901) at 1 h. The ensemble performance peaked at 2 h with an AUC of 0.869 (95% CI 0.789, 0.934) and then decreased at 3 h to an AUC of 0.807 (95% CI 0.717, 0.886). The performances of the models at different post-thaw time points are shown in Fig. 1.

### Embryologist performance and embryologist + model performance

At 2 h, the embryologists achieved an AUC of 0.829 (95% CI 0.747, 0.896) on average. The highest embryologist AUC at that time point was 0.851 (95% CI 0.764, 0.923), and the lowest embryologist AUC was 0.814 (95% CI 0.714, 0.887). With augmentation, on average, embryologist performance increased to 0.880 (95% CI 0.801, 0.936), a statistically significant change in AUC of 0.051 (95% CI 0.021, 0.083). The greatest AUC increase for a single embryologist was 0.067 (95% CI 0.028, 0.110), and the lowest increase was 0.033 (95% CI 0.001, 0.067). All embryologists had a statistically significant improvement in performance with augmentation at 2 h.

**Figure 2:**
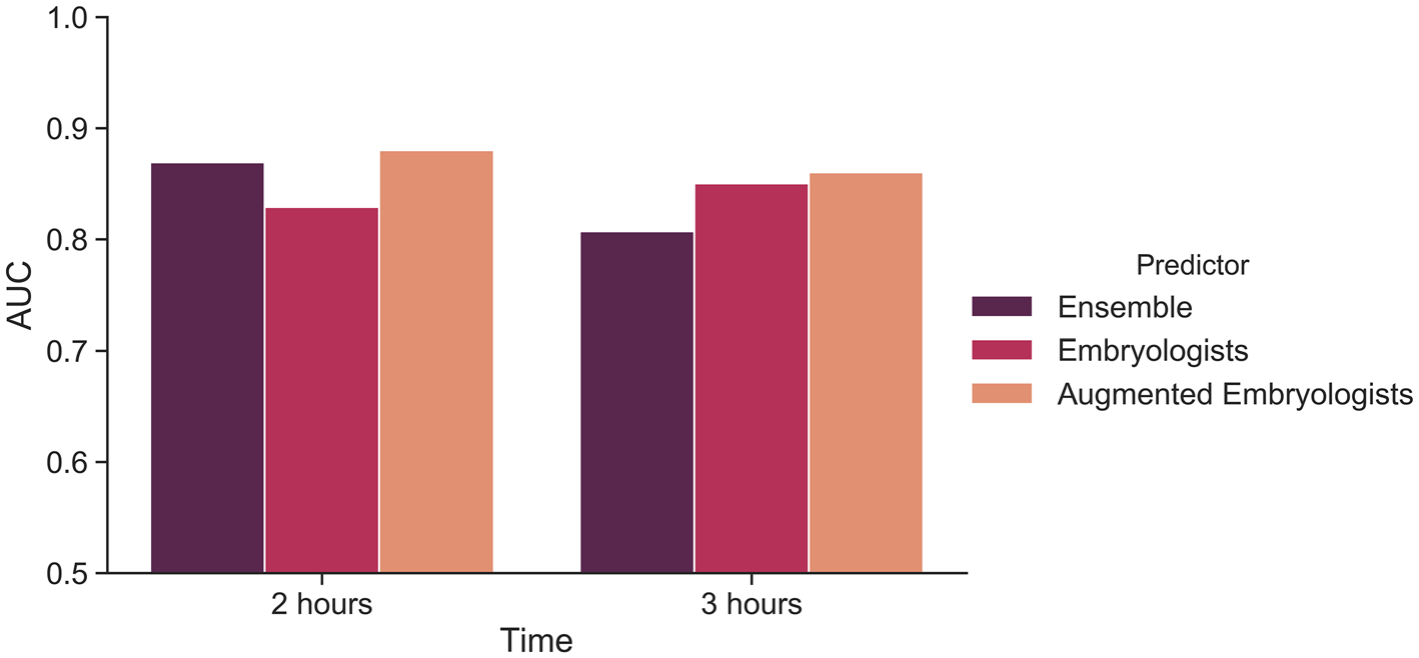
Model and embryologist average performance. Performance of the ensemble model, average embryologists and average augmented Embryologists at 2 h and 3 h post-thaw.

At 3 h, the embryologists achieved an AUC of 0.850 (95% CI 0.773, 0.908) on average. The highest embryologist AUC at that time point was 0.883 (95% CI 0.805, 0.938), and the lowest embryologist AUC was 0.815 (95% CI 0.72, 0.888). With augmentation, on average, embryologist performance increased to 0.860 (95% CI 0.783, 0.922), though this change by an AUC of 0.01 (95% CI −0.018, 0.037) was not statistically significant. No embryologist had a statistically significant improvement in AUC with augmentation at 3 h. The average AUCs for the embryologists with and without augmentation, in addition to the AUCs for the ensemble model at 2 h and 3 h, are reported in Fig. 2. The individual AUC of each embryologist, augmented embryologist, and improvement are reported in Table 1.

**Table 1:** Individual embryologist performance.

	**Embryologists** **(95% CI)**	**Embryologists + model (95% CI)**	**Improvement** **(95% CI)**
**2 h**			
Junior 1	0.83 (0.746, 0.905)	0.879 (0.797, 0.94)	0.05 (0.013, 0.088)
Senior 1	0.851 (0.764, 0.923)	0.884 (0.807, 0.942)	0.033 (0.001, 0.067)
Junior 2	0.82 (0.729, 0.899)	0.887 (0.809, 0.949)	0.067 (0.028, 0.11)
Senior 2	0.814 (0.714, 0.887)	0.869 (0.783, 0.932)	0.055 (0.019, 0.096)
Average	0.829 (0.747, 0.896)	0.880 (0.801, 0.936)	0.051 (0.021, 0.083)
**3 h**			
Junior 1	0.815 (0.72, 0.888)	0.840 (0.751, 0.909)	0.024 (−0.013, 0.062)
Senior 1	0.878 (0.8, 0.94)	0.875 (0.794, 0.938)	−0.003 (−0.033, 0.026)
Junior 2	0.825 (0.741, 0.899)	0.841 (0.755, 0.913)	0.015 (−0.014, 0.046)
Senior 2	0.883 (0.805, 0.938)	0.885 (0.808, 0.943)	0.002 (−0.027, 0.033)
Average	0.850 (0.773, 0.908)	0.860 (0.783, 0.922)	0.010 (−0.018, 0.037)

Performance of the embryologists and embryologists + model (AUROC) reported with the improvement with augmentation.

## Discussion

In this study, we developed a deep learning model that utilizes time-lapse images of freeze–thaw blastocysts during their first 3 h post-thaw to predict survival after a total of 25 h post-thaw in culture. Our data show these predictions can be combined with embryologist predictions to improve survival prediction. At 2 h post-thaw, augmenting embryologist predictions using the model resulted in a significant improvement in the average embryologist performance. Equivalence in average embryologist performance was noted with model augmentation at 3 h post-thaw. Even if equivalent, the model allows for reproducible predictions that will streamline lab processes and allow the lab team members to ascertain if embryos did not survive so that a clinical decision on next steps (secondary thaw, etc.) can be expedited. To our knowledge, this is the first proof of concept that deep learning model to evaluate in culture survival of embryos after thaw.

Observations have long been used to help identify embryo viability. Static grading systems, such as the David Gardner grading system^14^, are often used to assess blastocyst quality, but these grading systems are more commonly applicable to fresh rather than freeze–thaw blastocysts^15^*.* Although assessing freeze–thaw cleavage embryos has also become a well standardized practice that predominantly uses blastomere morphology and survival as indicators of viability, this level of standardization is less apparent in the assessment of viability of freeze–thaw blastocysts^18–21^. Freeze–thaw blastocysts have a different morphology to fresh embryos and are graded using different metrics to those used for fresh embryos, such as the level of re-expansion of the thawed blastocyst to its original size. Additionally, embryologists often have different opinions regarding which blastocysts are more likely to survive transfer and implant successfully; this subjectivity produces inconsistencies in the embryo selection task^22,23^. With single embryo transfer being the standard of care and the increasing use of embryo cryopreservation, it is important to gain a better understanding of factors affecting survival post-thaw and develop a standardized practice for inferring viability of freeze–thaw blastocysts.

Several studies have used morphokinetics to predict blastocyst development or pregnancy. Wong et al. used time-lapse imaging to propose that early morphokinetic markers could determine development of freeze–thaw zygotes to the blastocyst stage, although these blastocysts were not transferred, and little was known about their survival^24^. Additionally, Kirkegaard et al. tested whether proposed predictors of high quality blastocysts could also determine pregnancy, and found that within 48 h of culture, certain morphokinetic features could predict development to high quality blastocyst, but only age could predict pregnancy outcomes^25^. Time lapse technology has also enriched the viability analysis of cleavage embryos, helping to predict development to blastocyst stage as well as implantation potential^22,23,26,27^**.** Rienzi et al. developed a logistic regression model to predict how well time of morulation and trophectoderm quality could determine the likelihood of a pregnancy after a single-embryo transfer, and demonstrated a statistically significant association between these two variables and live birth^28^. However, these methodologies required lengthy manual assessment of morphological and morphokinetic features and did not utilize deep learning algorithms to automatically capture complex patterns directly from images.

More recently, deep learning algorithms have been developed on time-lapse technology to predict embryo survival or quality, but these have been limited in application to fresh embryos. Tran et al. developed a model on 1835 unique time lapse-videos of fresh, cultured embryos up to day 5 from 1648 individual patients, and predicted pregnancy with fetal heartbeat, and obtained a test AUC of 0.93^29^. However, of the embryos they assessed, only 29% consisted of embryos that were cryopreserved and thawed for a later transfer, and there were no videos of the post-thaw blastocysts in the dataset. Khosravi et al. developed STORK, a deep learning model trained on 12,001 time-lapse images from 1764 embryos to predict good-quality and poor-quality embryos with 0.987 test AUC; however, their trained algorithm also did not consider freeze–thaw blastocysts^7^. In a recent retrospective cohort study, Coello and colleagues recently showed that embryo reexpansion of warmed blastocysts evaluated via time-lapse imaging is correlated to their implantation capability, but they did not compare their predictive capability to embryologists or use deep learning to evaluate embryos^30^. To the best of our knowledge, our study is the first to utilize a deep learning model on time lapse videos of freeze–thaw blastocysts to determine whether an embryo would survive the freeze–thaw process in vitro.

The model achieved the highest AUC when using frames at 2 h from time-lapse videos of post-thaw blastocysts compared to other time points. No annotation and manual assessment of blastocyst images was required from embryologists; the model used raw images as inputs for the prediction of likelihood of embryo survival at 25 h post-thaw. The data used to develop and validate the model consisted of raw frames extracted from the time-lapse videos as opposed to the entire videos themselves, with the intention that this setup could be directly applied in clinics for which time-lapse technology is unavailable and only blastocyst images could be obtained. Additionally, using images allowed independent predictions to be made at different time points, facilitating an understanding of the optimal time for assessment of the embryo without other temporal context**.**

It is important to note that this is a proof-of-concept study that needs further investigation prior to clinical use. The prediction of in vitro viability at 25 h post thaw must be correlated with in vivo clinical outcomes of transferred embryos, an undertaking which is underway. If this is proven, then the system can have large clinical implications in recognizing embryos with lower reproductive potential prior to thaw, and ultimately help increase pregnancy rates per transfer. Furthermore, the important decision of whether to still transfer embryos or discard embryos recognized as not having survived the thaw, as well as whether to initiate the thaw of another embryo, should be made by the provider in consultation with the embryology team.

There are several limitations to our study. First, we did not feed the entire freeze–thaw time lapse video into the model, but rather extracted frames and used the images instead. This would not allow the model to infer possible temporal patterns in blastocyst development post-thaw and its relationship to survival. Second, our data consisted entirely of retrospective videos and captured using a single camera, and it is therefore unclear how our model may generalize to time-lapse videos from different clinics, or those captured using different time-lapse incubator systems. Third, we used survival at 25 h post-thaw as a proxy for embryo survival post-thaw and this may not completely reflect the uterine environment *in-vivo*. Fourth, aneuploid embryos were used for this study. An ideal clinical corollary would be to use euploid embryos however, differential survival was observed. Fifth, in our augmentation experiment, we averaged the prediction of the model and that of the embryologist, but we did not explore how showing the model output to the embryologist may influence their prediction; such a study may lend further insight into a deep learning assistance setting^31–36^. Sixth, we performed the study retrospectively and with 4 embryologists, but a prospective non-selection study with a larger number of embryologists and data from multiple clinics would be required to demonstrate generalizability outside our single center. Seventh, the selection of patients with an equal amount of viable and non-viable blastocysts may create a biased dataset for our test set. Future work will focus on ensuring that dataset mimic as closely as possible the characteristics of patient embryos upon which decisions are made. Lastly, there was a difference in the average age of viable and non-viable embryos, which is expected as age is well documented to correlate with embryo quality. Since age was not included in the algorithm, this does not impact the validity of the study. That said, it does open an opportunity to include age and other patient characteristics into future models and evaluate whether predictions can be further improved.

In conclusion, the freeze/thaw injury of a blastocyst may not be initially apparent upon thaw. It is possible that some failures of pregnancy could be the result of such injury. More diagnostics may help to discern the immediate post-thaw survival from ongoing survival and therefore competence of the embryo to implant *in-vivo.* Our study investigated the extent to which deep learning models could predict the blastocyst surviving the freeze–thaw process to 25 h post-thaw in culture using different assessment points up to 3 h. Our study is the first to demonstrate that a deep convolutional neural network can be used and combined with embryologists’ assessments at 2 h post-thaw to improve predictions of survival 25 h post thaw over embryologists’ assessment alone. Our model, when validated prospectively with a non selection study of euploid embryos, may aid embryologists in ascertaining which embryos may have failed to survive the thaw process, increasing pregnancy rates per transfer by preventing the transfer of non-viable embryos.

## Methods

### Data

We designed the data collection to parallel the decision time points in a clinical workflow for FETs. Typically, an embryo would be thawed at time 0 h (0 h) and assessed for initial survival and transferred at 2 h (2 h) or 3 h (3 h) post-thaw. However, its sustained survival following this time-point cannot be evaluated in vivo given it has been transferred; only the pregnancy outcome is able to be evaluated. In our design, we maintained the thaw and assessment time of a clinical workflow and determined the sustained embryo survival in culture at 25 h (25 h) following the freeze–thaw process. All embryos were cultured in Global media + 10% LGPS (Life Global Protein Supplement), which is the standard for our center. Survival at 25 h post thaw was chosen as embryos not meeting criteria to freeze or transfer on day 5 and 6 are cultured an extra day in our center.

Our primary outcome was the survival of the embryo post-thaw at 25 h. In this study, survival at 25 h post-thaw was defined as re-expansion of the blastocoel cavity, minimal cell lysis, and pulsing with progressively larger blastocoel volume. Thawed embryos that did not re-expand, exhibited progressively smaller volumes while pulsing, or had lysis of more than 50% of cells at 25 h post-thaw were defined as having failed to survive.

The dataset consisted of 652 time-lapse videos of post-thaw blastocysts collected at the Center for Reproductive Health at University of California, San Francisco between January 2019 and May 2020 from 119 patients who volunteered to donate their embryos. Study embryos were previously biopsied and collapsed prior to cryopreservation, and consisted of chromosomally abnormal embryos donated to research. These disposition decisions were made in writing by patients prior to cryopreservation and genetic testing. Images were captured using the Embryoscope time-lapse system (Vitrolife, Sweden). Each video was recorded starting at embryo thaw, with a frame rate between 0.1 and 0.2 h. Videos were reviewed, then annotated by an embryologist with the binary survival outcome: label 1 represented survival, and label 0 represented failure to survive, and the survival of the embryo was determined by the embryologist by reviewing the complete timelapse video. The embryologist was blinded to the prediction of the algorithm or the other participating embryologists and evaluated the continuous development of the embryo. There were no censored observations in the dataset.

The dataset was split into training, validation, and test sets. The training set was used to optimize models, the validation set was used to compare and choose models, and the test set was used to evaluate the chosen models. Embryologists were evaluated using the test set so that both the algorithm and the embryologists were evaluated on the same data. The test sets and validation sets were first sampled such that they contained an approximately equal number of embryos that survived and those that did not. The remaining videos were included in the training set. There was no patient overlap among the videos split into each set.

In this work, we focus on building out the proof-of-concept by demonstrating the development of a deep learning algorithm and focusing on combining the predictions of the algorithm with the experts. It is important to note that saliency maps, such as those enabled by class activation mapping, would be possible future work that could enable characterization of model attention. This has not been the focus of this work, and as such, we are unable to evaluate which parts of the embryo image are being used by the computer to make recommendations. We further note that recently shown limitations of saliency maps call into question their trustworthiness as a decision aid^11,12^.

All methods were carried out in accordance with relevant guidelines and regulations. The study was approved by the UCSF institutional review board (IRB). Informed consent was obtained from all subjects for the use of their tissues in this study.

### Model development

The task for the deep learning algorithm was to predict embryo survival to 25 h using images of the embryo within the first 3 h. A deep learning model was trained to output the probability of survival to 25 h using a single image of an embryo taken at 0.5-h intervals post-thaw up to 3 h. In this study, a convolutional neural network, a particular type of deep learning model that is specially designed to handle image data, was used. Convolutional neural networks scan over an image to learn features from local structure and aggregate the local features to make a prediction on the full image. The parameters of each network were initialized with parameters from a network pretrained on ImageNet. The final fully connected layer of the pretrained network was replaced with a new fully connected layer producing a 2-dimensional output, after which the softmax function was applied. This is an activation function that outputs the probabilities of each potential outcome given a real-valued vector input, whereby larger values correspond to larger probabilities. In this instance, it is used to obtain the predicted probabilities of survival success and failure.

Models were trained, validated, and tested on frames from the same post-thaw time point. Before inputting the images into the network, the images were resized to 224 pixels by 224 pixels and normalized based on the mean and standard deviation (SD) of images in the ImageNet training set. Because the outcome is invariant to flipping and rotation transformations of the input, a random horizontal flip and random vertical flip was applied with 50% probability to each image in the training set before being fed into the model for data augmentation; a random rotation between 0 and 360 degrees was also applied.

All model variants were optimized on the binary cross-entropy loss using stochastic gradient descent with momentum to update the model’s parameters^13^. The learning rate was tuned for each model and strategy by selecting the learning rate among 5e−4, 1e−3, and 2e−3 which led to the lowest validation loss on the validation set. The optimal learning rate across all model architectures was 1e−3. For each model and training strategy, the momentum parameter was set to 0.9 and the dampening was set to 0. The momentum parameter is used to aggregate gradients at each iteration to facilitate convergence; in this instance, 90% of the previous gradient is aggregated with 10% of the current gradient. After every epoch (one pass of the training dataset), the model was evaluated on the validation set and the model was saved based on the best validation loss. To combat overfitting, L2 weight decay of 1e−4 was added to the loss for all trainable parameters. This works to decay larger parameters towards (but not exactly to) zero and reduce the chance of overfitting of the model.

Models were trained using four convolutional neural network architectures: ResNet18, ResNet34, ResNet50, and DenseNet121, widely used for medical image classification^14–17^. A non proprietary model ensemble, called *EmbryoNeXt*, was created by averaging the rank predictions of models trained with these different architectures, a common methodology applied in machine learning. All models were trained on NVIDIA GeForce GTX 1070 GPUs using the PyTorch library v1.4.0 using a batch size of 16 examples.

### Embryologist benchmarks and augmented embryologist

We compared the performance of EmbryoNeXt to that of expert embryologists. Four embryologists, two junior embryologists (2 years of experience), and 2 senior embryologists (8 + years of experience), graded the test set on a scale of 1 to 10 (with 0.5 increments) at times 2 h and 3 h. The 1–10 score was a composite based on cell survival (0–3), expansion (0–3), cohesion (0–2), and cellularity (0–2) weighted to favor expansion and cell survival. Each embryologist graded the likelihood of survival, examining all frames at 2 h first and then all frames at 3 h independently. They were blinded to the true survival outcomes, clinical histories, and patient identifiers.

**Figure 3:**
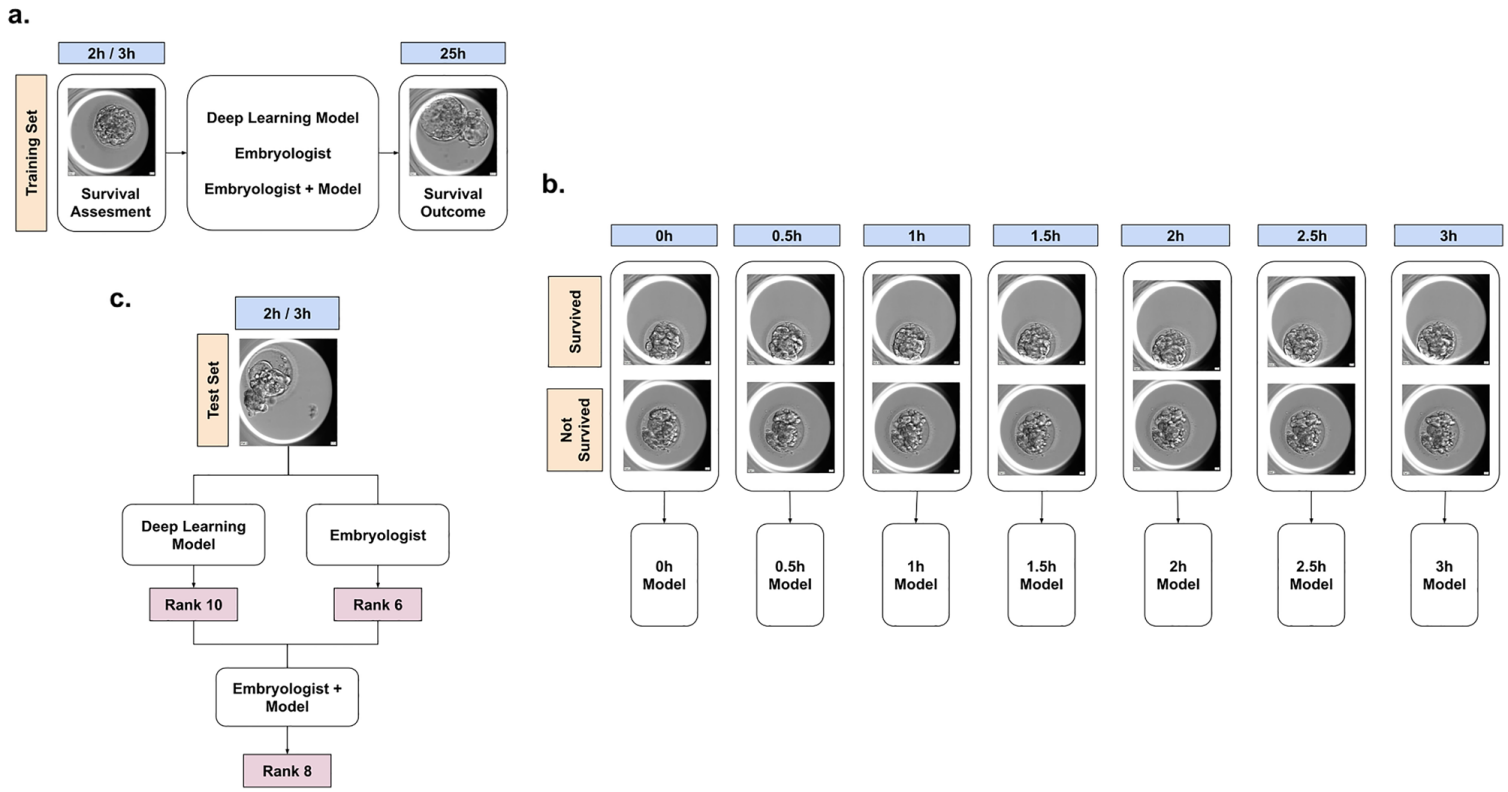
Schematic of the task, model training, and inference. (**a**) The task was to predict blastocyst survival at 25 h using an image of the blastocyst up to 3 h post-thaw. (**b**) At training time, frames at different time points up to 3 h were extracted from the video, and models were trained on both videos of survived and not survived embryos. (**c**) At inference time, the predictions of the model and embryologist were converted to prediction ranks, which were combined together to produce an augmented embryologist rank output.

We also combined the predictions of EmbryoNeXt with the predictions of each embryologist. In this setup, the embryologist was ‘augmented’ by averaging the rank predictions of EmbryoNeXt with the rank of the embryologist (converted from the composite score) on a particular example, relative to other examples in the test set. Rank predictions were used rather than the absolute probability outputs or embryologist scores since both model outputs and embryologist outputs may not be calibrated. A schematic of the setup, model training, and inference is detailed in Fig. 3.

### Statistical analysis

We compared the diagnostic performance of the models and the embryologists using the area under the receiver operating characteristic curve (AUC). To assess whether augmentation significantly changed the performance, we computed the difference in performance on the test set with and without augmentation. The nonparametric bootstrap was used to estimate the variability around each of the performance measures; 1000 bootstrap replicates from the test set were drawn, and each performance measure and difference was calculated for the model and the embryologist on these same 1000 bootstrap replicates. This produced a distribution for each estimate, and the 95% bootstrap percentile intervals were reported to assess significance at the *p* = 0.05 level. Model training and statistical analysis were performed using Python3.

## Data Availability

The datasets generated during and/or analysed during the current study are available from the corresponding author on reasonable request.
